# Association of *SIRT1* rs3758391 Polymorphism With T2DM in Bangladeshi Population: Evidence From a Case‐Control Study and Meta‐Analysis

**DOI:** 10.1002/hsr2.70495

**Published:** 2025-02-19

**Authors:** Rezwana Ahmed, Mushfikur Rahman Safa, Zahidul Islam Zahid, Md. Mazharul Islam Chowdhury, A.B.M. Kamrul Hasan, Md. Shaki Mostaid, Hasan Mahmud Reza

**Affiliations:** ^1^ Department of Pharmaceutical Sciences North South University Dhaka Bangladesh; ^2^ Appalachian College of Pharmacy Oakwood Georgia USA; ^3^ Department of Endocrinology Mymensingh Medical College Mymensingh Bangladesh

**Keywords:** Bangladesh, risk, rs3578391, SIRT1, type 2 diabetes mellitus

## Abstract

**Background and Aim:**

Type 2 diabetes mellitus (T2DM) remains one of the major causes of morbidity and mortality worldwide, including Bangladesh. SIRT1, an NAD‐dependent deacetylase, is involved in energy homeostasis and protects β‐cells of the pancreas from oxidative stress. Single nucleotide polymorphisms (SNPs) in the *SIRT1* gene have been found to be associated with T2DM in several populations, however, with conflicting results. The aim of this present case‐control study, along with the meta‐analysis, was to elucidate the association of rs3758391 polymorphism with the susceptibility to T2DM in Bangladeshi population.

**Methods:**

72 T2DM patients and 90 healthy controls were enrolled in our study and polymerase chain reaction‐restriction fragment length polymorphism (PCR‐RFLP) was employed for genotyping the SNP. Odds ratio (OR) with 95% confidence interval (95% CI) was used to represent the association of *SIRT1* polymorphism with T2DM. For the meta‐analysis six studies were included and pooled odds ratio with 95% CI were calculated for six genetic models using the random effects model. Heterogeneity and publication bias was also calculated for each study.

**Results:**

A significant association was found between rs3758391 polymorphism and increased risk of T2DM under codominant TT versus CC (OR = 3.88, 95% CI = 1.34–11.25, *p* = 0.012), recessive TT versus CC + CT (OR = 2.83, 95% CI = 1.12–7.09, *p* = 0.027) and allelic T versus C (OR = 1.67, 95% CI = 1.07–2.60, *p* = 0.024) genetic models. However, no significant association between rs3758391 and other biochemical and anthropometric parameters were found. Our meta‐analysis showed no statistically significant association of this polymorphism.

**Conclusion:**

We conclude that, polymorphism at rs3758391 of *SIRT1* gene conferred an increased risk of T2DM in Bangladeshi population.

## 1 Introduction

Among the noncommunicable diseases, diabetes mellitus (DM) remains one of the major causes of death and disabilities globally. About 529 million people were living with DM worldwide in the year 2021 and this figure is estimated to reach 1.31 billion by 2050 [1]. Type 2 diabetes mellitus (T2DM), forms the bulk of DM, which in 2021 accounted for 96% of the total DM prevalence [1]. T2DM is characterized by hyperglycemia, caused primarily by defects in insulin secretion by the β‐cells of the pancreas and resistance of the target tissues to the effects of insulin [2]. Inflammation, adipokine dysregulation, abnormalities in the gut microbiota, immune dysregulation and excessive reactive oxygen species generation serve as important pathophysiological factors in the development of T2DM [3, 4]. In addition, obesity, high‐calorie diet and sedentary lifestyle are the major factors that drive T2DM epidemic in this modern society [5, 6]. Notably, genetic factors play a significant role in the development of T2DM, and several genetic loci have been identified by genome wide‐association studies that increase the risk of T2DM [7, 8].

Sirtuins are a family of nicotinamide adenine dinucleotide (NAD)–dependent protein deacetylases [9, 10], and sirtuin 1 (SIRT1) is the most notable and studied one amongst them. Several proteins are the targets of SIRT1, such as those that are important in energy metabolism [11, 12, 13, 14], DNA repair [15], and inflammation [16]. SIRT1 is expressed in tissues that are important in metabolism such as the pancreas, liver, adipose tissue, heart, brain, and muscle [17]. It plays essential roles in nutrient‐sensing and insulin signaling pathways, including stress responses with consequent effects on cellular proliferation, survival, and apoptosis [18]. During calorie restriction, increased expression of SIRT1 occurs that contributes to delaying the onset of diseases of aging such as diabetes, atherosclerosis, and cancer [19, 20]. Accumulating evidence suggests that SIRT1 regulates glucose and lipid metabolism, as well as improving the sensitivity of the skeletal muscle, liver, and adipose tissues to the effects of insulin [21]. In addition, it exerts positive effects on pancreatic beta cells' function and mass [21], more specifically, it plays important roles in the glucose‐ATP signaling and insulin secretion from the pancreatic beta cells [22, 23]. Moreover, overexpression of SIRT1 or using SIRT1 activators in mice models improved glucose homeostasis and insulin sensitivity [24, 25, 26, 27]. Consequently, SIRT1 dysregulation has been implicated in diabetes and thereby, it may be considered as a therapeutic target for some debilitating diseases, for example, neurodegeneration, osteoarthritis, cardiovascular disease, and diabetic complications [17, 28, 29, 30, 31, 32, 33, 34]. Therefore, *SIRT1* serves as a plausible candidate gene that needs to be investigated to assess the relationship between *SIRT1* and susceptibility to T2DM. Furthermore, quite a few single nucleotide polymorphisms (SNPs) of the *SIRT1* gene have already been suggested to be involved in the increased risk of T2DM.

The location of the *SIRT1* gene is in chromosome 10q21.3, consisting of nine exons and eight introns [35]. The SNP rs3758391 resides in the promoter region of the *SIRT1* gene. Recently some studies have reported the effect of rs3758391 SNP on T2DM susceptibility, however, the results have been largely inconsistent. Sadeghi et al. reported that polymorphism of rs3758391 conferred a protective effect for T2DM in the Iranian population [36], whereas, an increased risk was reported by Faradonbeh et al. and Cruz et al. in the Iranian and Mexican population, respectively [37, 38]. However, no significant association was found between rs3758391 and susceptibility to T2DM in the Chinese population by Peng et al. [39]. This finding was further corroborated by two studies in Indian and Finnish population where they also reported negative association for this polymorphism [40, 41]. Hence, under these circumstances, we aimed to evaluate the association between *SIRT1* rs3758391 polymorphism and T2DM in a sample of Bangladeshi population and assess how our findings compare with the previous results. Moreover, we assessed the correlation of the SNP with the clinical parameters. In addition, we performed a meta‐analysis of the previous studies and compared it with our findings to further investigate the association of this SNP with T2DM.

## 2 Materials and Methods

### 2.1 Study Subjects and Anthropometric Measurements

This case‐control study consisted of 72 T2DM patients and 90 healthy controls. All the patients were recruited from different specialized diabetic hospitals in Dhaka city between November 2021 and January 2023. An expert endocrinologist at the concerned center confirmed the diabetes diagnosis according to the International Diabetes Federation (IDF) criteria, that is, fasting blood glucose concentration ≥ 6.1 mmol/l or HbA1c ≥ 6.5%. Age and gender matched healthy controls were recruited and they were confirmed of having no T2DM by the same diagnostic procedures as the cases. Exclusion criteria for the study subjects were as follows: pregnant and lactating women, subjects with T1DM, prediabetes and other endocrine dysfunctions. In addition, subjects with chronic diseases such as chronic liver disease, kidney disease unrelated to T2DM, autoimmune diseases or cancer were also excluded. The G*Power software was used to perform the post‐hoc power calculation [42]. Using the z‐test and logistic regression for a sample size of 162, odds ratio of 3.88, α error = 0.05 and binomial distribution, our study achieved a power (1‐β) = 97.6% with a critical z = 1.96. Ethical approval for the study protocol was obtained from the ethical review committee at North South University (#2020/OR‐NSU/IRB/1202). Before participation in the study, all study participants signed the informed consent form. Anthropometric data were collected from their medical records and personal interview.

### 2.2 Blood Sample Collection, Biochemical Measurements, and DNA Extraction

A total of 5 mL of venous blood was collected from the study participants into K‐EDTA tubes and the blood was stored at −80°C until further analysis was done. The biochemical analyses such as high‐density lipoprotein (HDL), low‐density lipoprotein (LDL), triglyceride (TG), total cholesterol (TC), and glycemic indices (glycated hemoglobin, HbA1c, fasting blood glucose, FBG; postprandial blood glucose, PPG) were performed using Humalyzer 3000 semi‐automatic biochemistry analyzer using the specific kit protocols at the North South University pathology center.

Genomic DNA was extracted from 200 µl of whole blood using a blood genomic DNA extraction kit (Favorgen, Taiwan) following the manufacturer's instructions. The purity and concentration of the extracted DNA was analyzed using NanoDrop microvolume spectrophotometer (Thermo Fisher Scientific, USA), with the absorbance ratio set at 260/280 nm. The DNA extracted was stored at −20°C until further analysis was done.

### 2.3 Genotyping Using Restriction Fragment Length Polymorphism (RFLP) Technique

**Figure 1:**
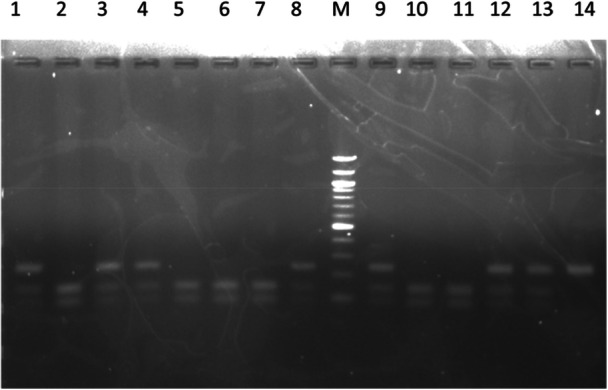
Restriction fragment length polymorphism analysis of rs3758391 C/T single‐nucleotide polymorphism (SNP) using NlaIII enzyme. 100‐bp ladder: Lane M; CC (241 bp): Lane14; TT (148 bp and 93 bp): Lane 2, 5, 6, 7, 10, 11; CT (241 bp, 148 bp, and 93 bp): Lane 1, 3, 4, 8, 9, 12, 13.

The genotyping of the rs3758391 polymorphism was performed by utilizing the polymerase chain reaction‐restriction fragment length polymorphism (PCR‐RFLP) technique. The PCR primers had the following sequence, Forward: 5′‐GTCACGCAGGTAATTGATGCAG‐3′ and Reverse: 5′‐GGCTTAGTGGAAAGCCCTTC‐3′, according to a previous study [36]. PCR was performed in a 20 µL reaction volume consisting of 100 ng of the DNA sample, 1 μL of forward and 1 μL of reverse primers, 10 μL of 2x PCR Mastermix (New England Biolabs, USA) and nuclease‐free water up to 20 μL. The conditions for the reactions were: initial denaturation for 3 min at 94°C followed by 30 cycles of denaturation at 94°C for 30 s, annealing at 57°C for 30 s, and extension at 72°C for 30 s, followed by a final extension for 5 min at 72°C. The PCR amplicons were analyzed on 1.5% agarose gels containing ethidium bromide to ensure the correct amplicon size of 241 bp. After confirmation of the desired amplicon size, 10 μL of the PCR product was digested with the restriction enzyme NlaIII (New England Biolabs, USA) at 37°C for 1 h. The digested products were visualized on a 2% agarose gel stained with ethidium bromide. A DNA ladder of 100 bp was utilized for the size estimation of the DNA fragments. The TT genotype yielded bands of 148 bp and 93 bp, the CC genotype yielded a band of 241 bp, while the CT genotype yielded bands of 241 bp, 148 bp, and 93 bp (Figure 1).

### 2.4 Meta‐Analysis Methodology

#### 2.4.1 Identification of the Relevant Studies

**Figure 2:**
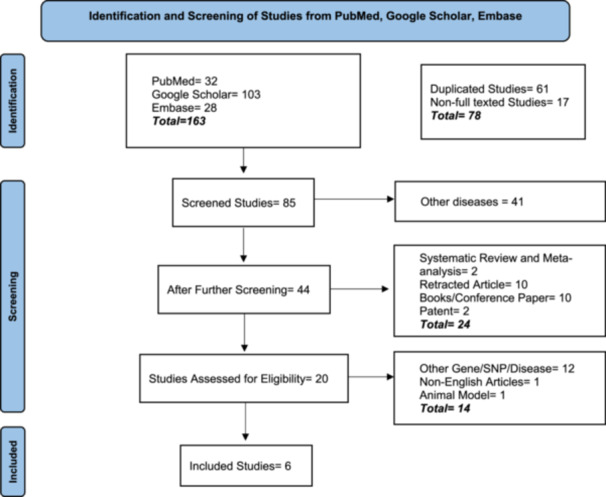
PRISMA‐P flowchart showing the selection of included studies. The initial search results were 163 articles (PubMed = 32; Google Scholar = 103; Embase = 28). After a thorough screening, data from six articles were selected.

PubMed, Google Scholar, and Embase was searched comprehensively to find relevant articles related to rs3758391 gene polymorphism and T2DM using keywords: “SIRT1,” “type II diabetes,” “rs3758391,” “type 2 diabetes,” “polymorphism,” “risk,” “association,” (Table 1). We performed the meta‐analysis using the PRISMA‐P guidelines [43], (Figure 2).

**Table 1:** Search query and found initial articles.

Website	Search query	Articles found
PubMed	“SIRT1,” “type II diabetes,” “rs3758391,” “type 2 diabetes,” “polymorphism,” “risk,” “association”	32
Google Scholar	“SIRT1,” “type II diabetes,” “rs3758391,” “type 2 diabetes,” “polymorphism,” “risk,” “association”	103
Embase	“SIRT1,” “type II diabetes,” “rs3758391,” “type 2 diabetes,” “polymorphism,” “risk,” “association”	28

#### 2.4.2 Inclusion and Exclusion Criteria

The inclusion criteria for the studies were: (1) investigating the association between polymorphism at rs3758391 of *SIRT1* gene with T2DM; (2) it must be a case‐control study with genotype counts. The review articles, studies with animal models, and studies investigating the association between other SNPs of *SIRT1* and T2DM were excluded (Figure 2).

### 2.5 Data Extraction Procedure

Two authors independently searched the databases to find the relevant articles and any discrepancy was resolved by discussion. The data included the relevant author's name, year, number of case and controls, followed by genotype data for the case and controls for each study (Table 2). The quality evaluation of the included studies for the meta‐analysis was assessed using the Newcastle‐Ottawa scale (Table 3).

**Table 2:** Characterization of included studies.

rs3758391													
Author	Year	Population		Allele (Case)		Allele (Control)		Genotype (Case)			Genotype (Control)		
		Case	Control	T allele	C allele	T allele	C allele	T/T	T/C	C/C	T/T	T/C	C/C
M. Cruz	2010	520	547	668	372	638	456	215	238	67	186	266	95
Ekta Rai	2012	692	850	686	698	866	834	173	340	179	238	390	222
Leena Kovanen	2015	300	5133	233	365	3992	6252	45	143	111	785	2422	1915
Yi Peng	2018	148	148	250	46	235	61	105	40	3	93	49	6
Ramin Tavakoli Faradonbeh	2019	132	66	121	143	27	105	35	51	46	6	15	45
Mohammad Bagher Sadeghi	2021	403	410	311	495	343	477	53	205	145	46	251	113
**Total**		**2195**	**7154**										

**Table 3:** Quality assessment of the included articles using Newcastle–Ottawa scale.

Author	Year	Selection				Comparability	Exposure			Total
		1	2	3	4	5	6	7	8	
M. Cruz	2010	*	*	*	*	*	*	*		*******
Ekta Rai	2012	*	*	*		*		*		*****
Leena Kovanen	2015	*	*	*		*		*		*****
Yi Peng	2018	*	*	*	*	*		*		******
Ramin Tavakoli Faradonbeh	2019	*	*	*	*	*		*		******
Mohammad Bagher Sadeghi	2021	*	*	*	*	*		*		******

1 = Adequate case definition

2 = Representativeness of the cases

3 = Selection of controls

4 = Definition of controls

5 = Comparability of cases and controls on the basis of the design or analysis

6 = Ascertainment of exposure

7 = Same method of ascertainment for cases and controls

8 = Nonresponse rate

### 2.6 Statistical Analysis

Two tailed tests were utilized for performing all the statistical tests. Normality distribution of data was checked using Q–Q plots and Shapiro‐Wilk tests. For not normally distributed data we used Mann‐Whitney U test for all continuous variables while we used the chi‐square test to compare the categorical variables between two groups. Multivariate logistic regression analysis was used to calculate the odds ratio (OR) along with 95% confidence intervals (CIs) to compare the genotype and allele frequencies between the case and control group. *p* < 0.05 was considered as statistically significant. The statistical analyses were performed using SPSS v27.

To conduct the meta‐analysis data was extracted from each case‐control study and organized in a 2 × 2 table. Classification criteria were diagnosis and type of alley they had. The pooled odds ratio (OR) with 95% CIs were calculated using the random effects model for all six genetic models to find the association of rs3758391 polymorphism with T2DM. Heterogeneity was calculated using Cochran's Q‐test and its magnitude was evaluated using I^2^ test. As we found significant heterogeneity (more than 50%) so we used the random effect model instead of the fixed effect model. Publication bias was evaluated using Begg‐Mazumdar's rank correlation test [44] and Egger's regression test [45]. Funnel plots were used to adjust the odds ratio in case of significant publication bias. Trim and fill method was used to perform the sensitivity analysis. We could not perform moderator analysis for ethnicity as the number of studies were limited. All the *p*‐values reported were two tailed and *p* < 0.05 was considered statistically significant. Meta‐analysis was performed using Comprehensive meta‐analysis version 3 [46].

## 3 Results

### 3.1 Characteristics of the Study Population

The anthropometric and biochemical measurements of the study subjects are summarized in Table 4. 162 subjects were included in this study, of whom 72 were patients with T2DM and 90 were healthy control subjects. No significant difference was found in the age of the subjects, between the T2DM and control groups, (*p* = 0.667). In case of the biochemical measurements, significant differences were found in HbA1c (*p* < 0.001), FBG (*p* < 0.001), PPG (*p* < 0.001), HDL (*p* < 0.001), and LDL (*p* < 0.001) between the T2DM patients and controls. However, no statistically significant difference was observed for SBP, DBP, TC, and TG between the cases and controls.

**Table 4:** Anthropometric and clinical parameters of T2DM patients and healthy controls.

Parameters	T2DM, median (IQR)	Controls, median (IQR)	*p* value
Age	47.34 (39.25–57.00)	48.27 (37.73–55.15)	0.667
Gender (male/female)	25/47	69/21	—
BMI (kg/m^2^)	24.10 (22.45–25.70)	24.60 (22.18–27.30)	0.187
SBP (mm Hg)	125.00 (120.00–130.00)	121.50 (120.00–130.00)	0.352
DBP mmHg	80.00 (80.00–90.00)	78.50 (60.00–80.00)	0.054
HbA1c (%)	8.43 (7.23–10.38)	4.70 (4.10–5.10)	**< 0.001**
FBG (mmol/L)	10.60 (8.33–14.00)	4.90 (4.48–5.20)	**< 0.001**
PPG (mmol/L)	16.40 (12.53–20.68)	6.30 (5.90–6.70)	**< 0.001**
TC (mg/dl)	192.50 (162.00–222.75)	195.00 (159.75–224.25)	0.874
HDL (mg/dl)	41.50 (35.00–52.00)	62.00 (51.00–74.50)	**< 0.001**
LDL (mg/dl)	122.00 (85.25–157.00)	86.50 (65.50–107.25)	**< 0.001**
TG (mg/dl)	168.50 (117.25–218.75)	186.50 (138.75–268.25)	0.516

*Note:* The Mann‐Whitney *U* test was used to compare the continuous variables between the T2DM and control groups. *p* < 0.05 was considered statistically significant. *p *< 0.05 values are made bold.

Abbreviations: BMI, body mass index; DBP, diastolic blood pressure; FBG, fasting blood glucose; HbA1c, glycated hemoglobin; HDL, high density lipoprotein; IQR, interquartile range; LDL, low density lipoprotein; PPG, postprandial blood glucose; SBP, systolic blood pressure; TC, total cholesterol; TG, triglycerides.

### 3.2 Genetic Association Analysis

The genotype and allele frequencies of rs3758391 polymorphism of the T2DM patients and healthy controls are shown in Table 5. We found that the codominant model (TT vs. CC; OR = 3.88; 95% CI = 1.34–11.25; *p* = 0.012) and recessive model (TT vs. CC + CT; OR = 2.83; 95% CI 1.12–7.09; *p* = 0.027), showed a significant association between rs3758391 polymorphism and risk of T2DM. We also found that the T allele also conferred an increased risk to T2DM by 1.67‐folds (T vs. C; OR = 1.67; 95% CI 1.07–2.60; *p* = 0.024). However, no significant association was found between the anthropometric‐biochemical measurements and CT + TT versus CC genetic model in either T2DM or controls (Table 6).

**Table 5:** Genotype and allelic frequencies of *SIRT1* rs3758391 polymorphism in T2DM patients and healthy controls.

Genotype	T2DM, *n* (%)	Control, *n* (%)	OR (95%CI)	*p*‐value
**Codominant**				
TT	7 (9.7%)	21 (23.3%)	3.88 (1.34–11.25)	**0.012** *
CT	43 (59.7%)	52 (57.8%)	1.57 (0.74–3.32)	0.242
CC	22 (30.6%)	17 (18.9%)	1 (reference)	
**Dominant**				
CT + TT	50 (69.4%)	73 (81.1%)	1.89 (0.91–3.91)	0.087
CC	22 (30.6%)	17 (18.9%)	1 (reference)	
**Recessive**				
TT	7 (9.7%)	21 (23.3%)	2.83 (1.12–7.09)	**0.027** *
CC + CT	65 (90.3%)	69 (76.7%)	1 (reference)	
**Overdominant**				
CT	43 (59.7%)	52 (57.8%)	0.92 (0.49–1.73)	0.803
CC + TT	29 (40.3%)	38 (42.2%)	1 (reference)	
**Alleles**				
T	57 (39.6%)	94 (52.2%)	1.67 (1.07–2.60)	**0.024** *
C	87 (60.4%)	86 (47.8%)	1 (reference)	

*Note:* The Chi‐square test was used to analyze the genotypes and allele frequencies in the groups. Binary logistic regression were used to assess the association between the genotypes and T2DM. *p* < 0.05 was considered statistically significant.

Abbreviations: CI, confidence interval; OR, odds ratio; T2DM, type 2 diabetes mellitus.

* *p* < 0.05.

**Table 6:** Association between *SIRT1* rs3758391 polymorphism and anthropometric and clinical characteristics of T2DM patients and healthy controls.

rs3758391	Genotype	BMI (kg/m^2^)	SBP (mm Hg)	DBP mmHg	HbA1c (%)	FBG (mmol/L)	PPG (mmol/L)	TC (mg/dl)	HDL (mg/dl)	LDL (mg/dl)	TG (mg/dl)
T2DM	CT + TT	24.15 (21.75–25.75)	125.00 (120.00– 130.00)	80.00 (80.00– 85.00)	8.65 (7.20–0.33)	10.45 (8.38–3.88)	16.90 (13.00– 20.35)	192.50 (164.25–216.75)	40.50 (35.00– 52.00)	122.00 (86.00–157.00)	175.50 (127.00– 216.75)
	CC (ref)	23.90 (22.63– 25.60)	130.00 (120.00–130.00)	80.00 (80.00– 90.00)	8.43 (7.30– 10.63)	10.80 (8.18–14.25)	15.95 (11.78– 20.88)	195.00 (147.00 –223.50)	42.50 (35.00–52.00)	106.00 (66.00–158.50)	148.50 (110.50– 226.50)
	*p* value	0.921	0.680	0.317	0.798	0.921	0.798	0.798	0.798	0.955	0.443
Control	CT + TT	24.60 (21.95– 27.65)	122.00 (120.00– 130.00)	80.00 (68.00– 80.00)	4.60 (4.15– 5.00)	4.90 (4.30– 5.20)	6.30 (5.85– 6.70)	200.00 (162.50– 229.00)	62.00 (51.00– 76.50)	88.00 (65.00– 108.50)	204.00 (145.00– 272.00)
	CC (ref)	24.60 (23.05– 25.15)	120.00 (110.00– 130.00)	70.00 (59.00– 80.00)	4.70 (3.85–5.30)	5.00 (4.80– 5.40)	6.35 (6.10– 6.78)	182.00 (144.50– 219.17)	63.00 (56.00– 69.50)	84.00 (60.50– 100.00)	158.93 (89.00– 255.00)
	*p* value	0.501	0.590	0.106	0.942	0.205	0.821	0.157	0.919	0.281	0.590

*Note:* The Mann‐Whitney test was used to assess the association between the studied genotype and the anthropometric and clinical data. *p* < 0.05 was considered statistically significant.

Abbreviations: BMI, body mass index; DBP, diastolic blood pressure; FBG, fasting blood glucose; HbA1c, glycated hemoglobin; HDL, high density lipoprotein; LDL, low density lipoprotein; PPG, postprandial blood glucose; SBP, systolic blood pressure; TC, total cholesterol; TG, triglycerides.

### 3.3 Meta‐Analysis Results

**Figure 3:**
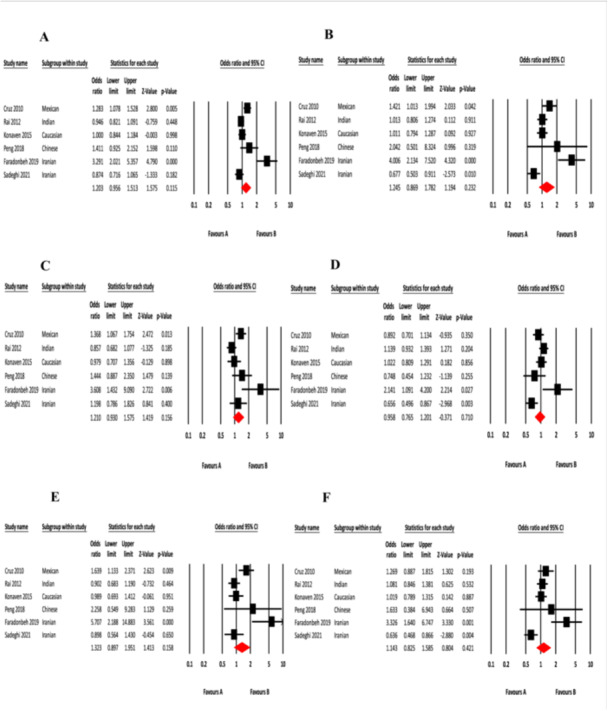
Forest plots of rs3758391 with T2DM risk in six genetic models. (A) Allelic model (T vs. C); (B) dominant model (CT + TT vs. CC); (C) recessive model (TT vs. CC + CT); (D) overdominant model (CT vs. CC + TT); (E) codominant 1 model (TT vs. CC) and (F) codominant 2 model (CT vs. CC).

**Figure 4:**
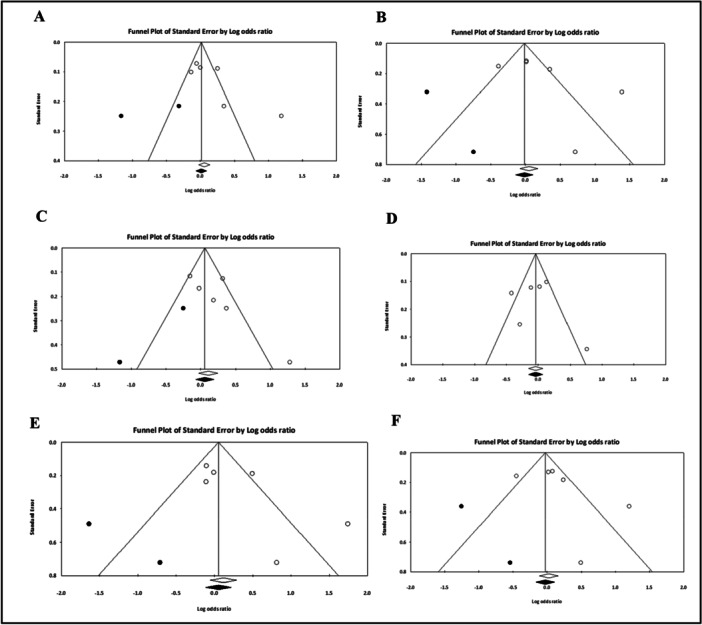
Funnel plots of rs3758391 with T2DM risk in six genetic models. (A) Allelic model (T vs. C); (B) dominant model (CT + TT vs. CC); (C) recessive model (TT vs. CC + CT); (D) overdominant model (CT vs. CC + TT); (E) codominant 1 model (TT vs. CC) and (F) codominant 2 model (CT vs. CC).

Our meta‐analysis revealed an increased odds ratio for T2DM in all six genetic models for this polymorphism although none of them were statistically significant (Table 7, Figures 3, 4). The highest odds ratio was found in the codominant model 1 (TT vs. CC; OR = 1.32, 95%CI = 0.90–1.95, *p* = 0.158), while the *p*‐value was lowest for the allelic model (T vs. C; OR = 1.20, 95% CI = 0.96–1.51, *p* = 0.115). We found a higher percentage of heterogeneity in all six models which reveals the variability of data in the case‐control studies which were included in this meta‐analysis. We found no publication bias in both Egger's regression test and Begg‐Mazumdar's rank correlation tests.

**Table 7:** Association of rs3758391 with type 2 diabetes.

Model name	Association test				Heterogeneity test			Publication Bias (*p* value)	
	Odds Ratio (OR)	95% CI	*p* value	Model	Q value	I^2^ value	*p* value	Egger's test	Begg‐Mazumdar's test
Allelic (T vs. C)	1.20	0.96–1.51	0.115	Rndom	33.68	85.15	0.00	0.10	0.19
Dominant (CT + TT vs. CC)	1.25	0.87–1.78	0.232	Random	29.87	83.26	0.00	0.22	0.19
Recessive (TT vs. CC + CT)	1.21	0.93–1.58	0.156	Random	15.73	68.21	0.01	0.13	0.09
Overdominant (CT vs. CC + TT)	1.20	0.77–1.20	0.710	Random	17.01	70.60	0.01	0.88	0.57
Codominant 1 (TT vs. CC)	1.32	0.90–1.95	0.158	Random	19.80	74.74	0.01	0.14	0.19
Codominant 2(CT vs. CC)	1.14	0.83–1.59	0.421	Random	21.81	77.07	0.01	0.31	0.35

## 4 Discussion

This study aimed to investigate the association between *SIRT1* rs3758391 polymorphism and T2DM in Bangladeshi population. We found that the SNP rs3758391 T > C conferred an increased risk to T2DM. The TT genotype increases the risk of T2DM by 3.88 and 2.83 times under the codominant model and recessive model, respectively. In addition, the T allele increased the risk of T2DM by 1.67 times. In addition, analysis of the clinical and anthropometric parameters between the patients and healthy controls based on their genotype as carriers and noncarriers revealed no statistical significance. This implies that the risk alleles have no impact on the hypertension markers, diabetes markers and total lipid profile. Again, the meta‐analysis showed that rs3758391 increased the risk of T2DM in all six genetic models. However, they were not statistically significant, likely due to the variability in the data of the included studies. Some of the previous studies are also in agreement with our present findings. In the study by Cruz et al. in the Mexican population, the T allele of rs3758391 was found to be associated with a higher risk of T2DM [38]. Fardonbeh et al. found that in Iranian population, the T allele of rs3758391 was also strongly associated with T2DM [37]. However, Peng et al. [39] and Kovanen et al. [41] could not find any significant association between rs3758391 and T2DM susceptibility in Chinese and Finnish populations, whereas the Sadeghi et al. reported that rs3758391 variant conferred a protective role against T2DM in Iranian population [36]. The differences in results may be partially explained by the differences in ethnicity, and the genetic variations may lead to differential expression level of SIRT1 protein in population of different ethnicities, thus serving as either a protective or risk factor.

SIRT1 regulates the activity of a variety of transcription factors, for example, forkhead‐box transcription factors (FOXOs), peroxisome proliferator‐activated receptor γ (PPAR γ) and nuclear factor‐κB (NF‐κB) [47]. SIRT1, as a regulatory molecule impacts many vital biological processes such as glucose and lipid metabolism, inflammation, apoptosis, oxidative stress, cellular senescence and circadian rhythms [48, 49]. Importantly, SIRT1 protects pancreatic β‐cells from dysfunctions induced by oxidative stress and inflammatory cytokines, likely through suppression of NF‐kB signaling [50]. It activates insulin signaling in skeletal muscle cells [51] and has the capacity to decrease inflammation in adipose tissue and monocytes, thereby leading to the alleviation of insulin resistance and T2DM [52]. Therefore, changes in the expression and activity level of SIRT1 may lead to development of pathological conditions. SIRT1 levels have been found to decrease in diet‐induced obesity in rodents [53, 54]. In humans, it has been reported that reduced expression levels of SIRT1 in circulating monocytes correlates with insulin resistance and metabolic syndrome [55] and its levels also decrease in adipose tissue of obese subjects [56]. Notably, reduced SIRT1 levels were found in patients with T2DM [39, 57, 58].

Our meta‐analysis revealed no statistically significant association of the rs3758391 polymorphism at *SIRT1* gene with T2DM. As far as we are aware, this is the first meta‐analysis done between rs3758391 single‐nucleotide polymorphism and T2DM. It is also worth noting that, the number of studies included for our meta‐analysis was comparatively small. Moreover, the included studies consisted of population from different ethnicities, which have resulted in significant heterogeneity. Although, we tried to counter that using the random effects model, but the results of our meta‐analysis cannot be generalized for people of all ethnicities. This may be a reason for difference in the outcome of our case‐control study with the meta‐analysis. Furthermore, moderator analysis based on ethnicity could not be performed due to the unavailability of studies with similar ethnic backgrounds. As a result, we see the discrepancies that must be further investigated by conducting more case‐control studies between this SNP and T2DM in larger sample size and similar ethnic backgrounds to see if there is ethnic difference and whether this SNP confers risk or protective effect to T2DM in Bangladeshi population.

Despite the work we carried out, our study has some limitations, which needs to be acknowledged. Our sample size was relatively small which may not be sufficient to give a very robust genotype‐disease interaction. In addition, previous studies have found decreased levels of SIRT1 in T2DM patients. However, we could not check the gene or protein expression levels in our study subjects, which would otherwise demonstrate the effect of the SNP on the gene expression levels. These limitations should be addressed in future studies. As T2DM is increasing at an alarming rate in Bangladesh, the development of effective, yet affordable means are required to assess the risk of development of T2DM based on genetic variants in our population. Future studies that include other SNPs and genes with larger sample sizes will be performed to help establish a T2DM biobank for the Bangladeshi population. This in turn, can assist clinicians in prevention, early detection and proper management of T2DM, helping to decrease the burden of healthcare costs for our developing country.

## 5 Conclusion

Our current study shows that rs3758391 is associated with T2DM susceptibility. However, future replication studies in diverse ethnic groups with larger sample sizes should be performed to obtain a more robust and comprehensive association result.

## Author Contributions

**Rezwana Ahmed:** conceptualization, data curation, formal analysis, funding acquisition, methodology, visualization, writing–original draft. **Mushfikur Rahman Safa:** investigation, data curation. **Zahidul Islam Zahid:** investigation, project administration. **Md. Mazharul Islam Chowdhury:** software, investigation. **A.B.M. Kamrul Hasan:** resources, writing–review and editing, supervision. **Md Shaki Mostaid:** conceptualization, formal analysis, methodology, software, validation, visualization, writing–review and editing. **Hasan Mahmud Reza:** conceptualization, methodology, supervision, validation, visualization.

## Conflicts of Interest

The authors declare no conflicts of interest.

## Transparency Statement

The lead author Rezwana Ahmed, Hasan Mahmud Reza affirms that this manuscript is an honest, accurate, and transparent account of the study being reported; that no important aspects of the study have been omitted; and that any discrepancies from the study as planned (and, if relevant, registered) have been explained.

## Data Availability

All authors have read and approved the final version of the manuscript. Corresponding author had full access to all of the data in this study and takes complete responsibility for the integrity of the data and the accuracy of the data analysis. The authors confirm that the data supporting the findings of this study are available within the article. The data that support the findings of this study are available from the corresponding author upon reasonable request.
